# Foam density mapping via THz imaging

**DOI:** 10.1038/s41598-024-64856-1

**Published:** 2024-07-06

**Authors:** Ilaria Catapano, Sonia Zappia, Paolo Iaccarino, Rosa Scapaticci, Ernesto Di Maio, Lorenzo Crocco

**Affiliations:** 1 Institute for Electromagnetic Sensing of the Environment, National Research Council of Italy, 80124 Naples, Italy; 2 Dipartimento di Ingegneria Chimica, dei Materiali e della Produzione Industriale, University of Naples Federico II, 80125 Naples, Italy; 3 Scuola Superiore Meridionale, Largo San Marcellino 10, 80128 Naples, Italy

**Keywords:** THz imaging, Density, Plastic foams, Electrical and electronic engineering, Imaging techniques

## Abstract

Plastic foams, near-ubiquitous in everyday life and industry, show properties that depend primarily on density. Density measurement, although straightforward in principle, is not always easy. As such, while several methods are available, plastic foam industry is not yet supported with a standard technique that effectively enables to control density maps. To overcome this issue, this paper proposes Terahertz (THz) time-of-flight imaging using normal reflection measurements as a fast, relatively cheap, contactless, non-destructive and non-dangerous way to map plastic foam density, based on the expected relationship between density and refractive index. The approach is demonstrated in the case of polypropylene foams. First, the relationship between the estimated effective refractive index and the polypropylene foam density is derived by characterizing a set of carefully crafted samples having uniform density in the range 70–900 kg/m^3^. The obtained calibration curve subtends a linear relationship between the density and the refractive index in the range of interest. This relationship is validated against a set of test samples, whose estimated average densities are consistent with the nominal ones, with an absolute error lower than 10 kg/m^3^ and a percentage error on the estimate of 5%. Exploiting the calibration curve, it is possible to build quantitative images depicting the spatial distribution of the sample density. THz images are able to reveal the non-uniform density distribution of some samples, which cannot be appreciated from visual inspection. Finally, the complex spatial density pattern of a graded foam sample is characterized and quantitatively compared with the density map obtained via X-ray microscopy. The comparison confirms that the proposed THz approach successfully determines the density pattern with an accuracy and a spatial scale variability compliant with those commonly required for plastic foam density estimate.

## Introduction

Plastics are economical, lightweight, and durable materials that can be moulded into a variety of products with a wide range of applications^1^. A large component of the plastic industry is the plastic *foams* industry: the global foam plastic market size was evaluated at around USD 102 billion in 2021, and it is expected to reach around USD 135 billion while growing at a compound annual growth rate of 3.6% by 2031^2^.

Plastic foams consist of two phases: a solid polymer matrix and a gaseous phase^3^. Due to their porous structure (the porosity is even as large as 90–95%), plastic foams are used, among others, for packaging, thermal insulation, acoustic absorption, buoyancy and impact absorption. According to the classification given by Ashby^4^, density is the main characteristic that defines the mechanical and functional properties of plastic foams; Young’s modulus and strength have a quadratic dependence on the density, whereas thermal insulation exhibits a linear dependence, provided the structure is a closed-celled one^4^.

Typically, plastic foams have uniform density and morphology, but graded foams and foams with a 3D *density map* are gaining attention in science and various technical fields^5,6^. In both cases, understanding if a required density map is actually obtained after the production process is still an open technical issue. In fact, plastic foams are three-dimensional (3D) materials, and a surface characterization (e.g., via scanning electron microscopy) is not enough to quantitatively and precisely characterize the foamed material^7^. X-ray computed micro-tomography ($\mu$-CT) is the most used tool that allows a non-destructive quantitative 3D characterization of plastic foams^8,9^. However, the low X-ray absorption of the polymeric matrix and the thin-structured cell walls causes problems during the reconstruction of the real cellular structure, complicating the post-processing operations^10^. Moreover, X-ray is a costly and time-consuming technology^11^. An alternative technique is the neutron tomography (*N* CT): compared to $\mu$-CT, *N*-CT is based on the enhanced neutron attenuation of polymers (due to the high hydrogen content in their macromolecules) and therefore the contrast for such structures is in general higher^10^. However, large instruments and complex post-processing efforts are needed also in this case^11^. To our knowledge, it seems that the plastic foam industry is not supported with an effective technique that enables a routinely determination and/or control of the density maps.

THz are electromagnetic waves ranging from 0.1 to 10 THz (wavelength from 3 mm to 30 μm), which can penetrate a wide range of non-conducting materials, e.g., plastics, polymers, ceramics, wood, and glass^12^. Accordingly, they allow for a contactless, safe, and non-destructive characterization of these materials, not limited to the surface of the object under test but also to its interior. Typically, structures of thickness up to a few centimeters along the incidence direction can be investigated, providing information at millimeter scale. THz imaging and spectroscopy have been considered in many fields^13–17^, among which cultural heritage^18^, security^19^ and pharmaceutical applications^20,21^, only to cite a few examples. Plastic foams have been, also, characterized by using THz and the performed spectroscopy studies have shown that their properties change depending on the kind of foam, blowing agent and density^22^. As a novel contribute to the available literature, this paper investigates the use of THz imaging to obtain quantitative density maps of graded plastic foams, which are attracting a growing interest in the foam industry thanks to their peculiar mechanical properties directly depending on the designed 3D density map.

In the ever-growing body of literature, some studies considering the use of THz for density estimation are worth being mentioned. An early attempt to map density was pursued by Koch et al.^23^, who exploited transmission measurements and derived a calibration curve to relate the density of wood, as obtained by gravimetric-volumetric methods, with the THz absorption as measured for the known sample thickness. Wood density characterization via THz transmission measurements was also considered by Kashima et al.^24^, where a linear regression approach was proposed to predict density and moisture content from refractive indices and absorption coefficients obtained from THz transmission measurements. THz has been also considered for density and porosity measurements of pharmaceutical tablets^21,25^, as well as for imaging foam density and defects^26^. This latter paper uses reflection measurements and an external reference structure. However, to the best of our knowledge, THz imaging of foam specimens with non-uniform volumetric density distribution has never been explored before.

The approach we propose is based on time-of-flight (ToF) THz imaging. With respect to the literature^16,19,21–28^, which mainly consider (single) transmission measurements, the adopted approach requires that the sample under test is positioned on a flat metallic plane and scanned along the *x*–*y* plane in normal reflection mode (sometimes referred to as double transmission). For each measurement point, the reflected signal is processed to determine the effective refractive index of the sample along the signal path. Specifically, the estimated effective refractive index is an average parameter depending on the sample dielectric features along the signal propagation path (i.e., the z-axis in our configuration) and its dispersive behaviour in the frequency range of the THz probing pulse. As such, the estimated equivalent refractive index accounts for the density variations occurring across the sample below the surface and along its depth. Then, provided the relationship between effective refractive index and mass density is available, the approach delivers a 2*D* map that represents the spatial distribution of the density, in which each pixel encodes the average density along the third dimension. Although it is very simple, the proposed approach is an effective and widely applicable procedure to estimate the refractive index, and thus the density, of uniform or not specimens, without requiring a priori information about their point by point thickness, while avoiding optimization procedures. Effective processing tools are available to estimate refractive index and thickness simultaneously^27,28^ but they account for uniform sample and a (single) transmission measurement setup.

The approach is initially applied to characterize a set of uniform samples with variable nominal density values in order to establish the relationship between the effective refractive index and the density. The experimentally achieved calibration curve reveals a linear relationship between these two quantities within the range of interest. This relationship is in good agreement with that derived by using the medium theory given by Scheller et al.^29^ and it is also validated by using another set of samples, referred to as test samples. THz surveys of the test sample reveal that their measured average density agrees with the one predicted by the calibration curve. In addition, THz images show that some of these samples have a not-uniform refractive index distribution. Hence, by means of the calibration curve, a quantitative description of their non-uniform density pattern, invisible to visual inspection, is obtained. Finally, the approach is exploited to image the density pattern of a foam sample having a 3D optimized density map for the three-point bending load condition^6^. The density map achieved via THz is validated with the one resulting from X-Ray microscopy. To the best of our knowledge, a similar comparison has never been presented before and the obtained qualitative and quantitative coherence supports the usefulness of the proposed approach as a simple strategy to exploit THz technology for mapping volumetric density variations, thus answering an open issue in foam industry.

## Materials and methods

### THz time-of-flight imaging

**Figure 1:**
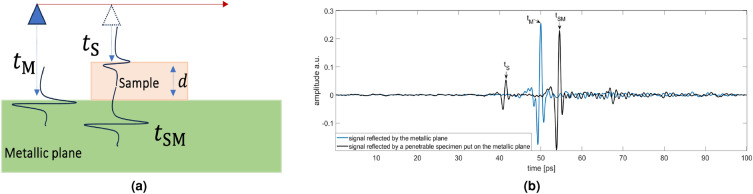
Measurement setup: (**a**) sketch of measurement procedure, (**b**) example of the measured signals referred to a metallic plane (blue curve) and a penetrable sample put on the metallic plane (black curve).

THz time-of-flight (ToF) reflection imaging allows the collection of a large amount of information about the sample under test. At each measurement point, the object is probed by a pulsed signal and the reflected waveform is collected within a certain observation time window. The reflected waveform exhibits only one peak due to the air-sample interface in the case of a not penetrable sample; otherwise, it accounts for the sample surface and its inner electromagnetic discontinuities. Specifically, if the sample is homogeneous and sufficiently thin to be entirely penetrated, the reflected waveform exhibits two peaks corresponding to the beginning (upper side) and the end (bottom side) of the sample along the wave propagation path, see Fig. 1. The time delay between these two peaks depends on the thickness of the sample and its electromagnetic properties (i.e., its refractive index). If the sample has a layered structure made up of electromagnetic different materials, the collected waveform appears as a pulse train where the temporal delay between successive pulses provides information about the sample stratigraphy. Therefore, a 3D image representing surface and interior of the sample can be obtained by scanning the whole sample^30^.

The ToF *t* is related to the distance *d* between the THz probes and the detected discontinuities as:

1 $$\begin{aligned} t = \frac{2d}{v} \end{aligned}$$

*v* = *c*/*n* being the electromagnetic wave propagation velocity into the medium wherein the propagation occurs, *c* the light propagation velocity and *n* the refractive index of the medium along the wave path.

In our set-up, the sample is positioned on a flat metallic plane and probed in normal reflection mode, i.e., the incidence angle is zero, see Fig. 1. Let $t_M$ and $t_{SM}$ denote the ToF referred to the metallic plane reflection when the signal propagates in air and when it crosses the sample, respectively, and let $t_{S}$ be the ToF related to the reflection due to the air-sample interface.

For each point $(x_p, y_p)$ belonging to the sample, the thickness, $\Delta (x_p, y_p)$ is readily estimated from Eq. (1), as:

2 $$\begin{aligned} \Delta (x_p, y_p) = \frac{c\Delta t_{SM}}{2}, \end{aligned}$$

where $\Delta t_{SM}=t_M-t_S(x_p, y_p)$ is the time-delay associated to the propagation that occurs in air (i.e., $n=1$). Hence, the map of $\Delta$ depicts the sample thickness, appraising variations whose size is equal or higher than the spatial range resolution of the adopted THz system.

Assuming an *effective medium* approximation for the sample under test, according to which the medium is approximated as a homogeneous and non-dispersive medium along the *z* direction. Each measurement point $(x_p, y_p)$ scanning the sample surface can be characterized by an *effective refractive index*, say $n_{eff}(x_p,y_p)$, obtained from Eqs. (1) and (2) as:

3 $$\begin{aligned} n_{eff}(x_p,y_p) = \frac{t_{SM}(x_p,y_p)-t_{s}(x_p,y_p)}{t_{M}-t_{S}(x_p,y_p)}. \end{aligned}$$

Accordingly, the plot of $n_{eff}$ provides a 2D quantitative map of the refractive index along the scanning surface with a spatial resolution depending on the measurement offset, which takes into account point-by-point variations of the sample thickness.

### Samples preparation

Among possible plastic foams, this study has been focused on Polypropylene (PP) foams. Two types of PP foams samples were produced:

- a set of *cylindrical samples* having a nominally uniform density in the range 70–900 kg/m^3^;
- a *graded-density sample*, a beam with a 3D density map optimally shaped for three point bending.

**Figure 2:**
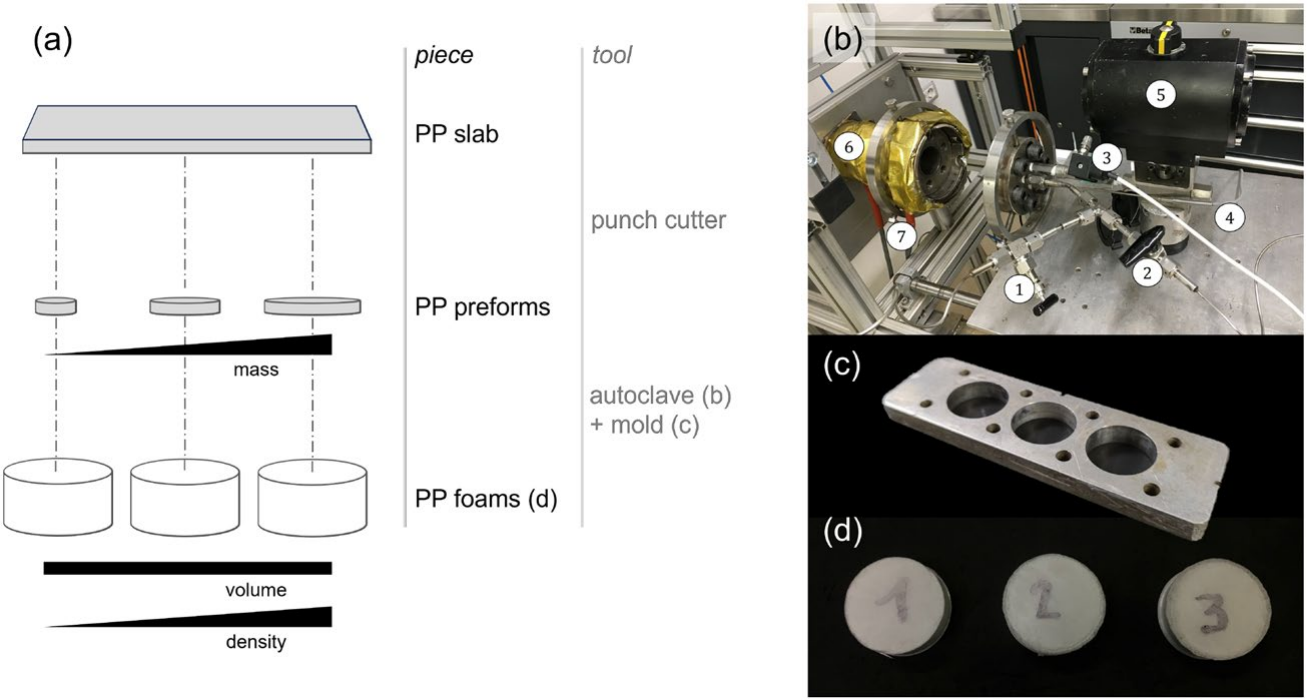
(**a**) Foam production process schematic. (**b**) Batch foaming apparatus: $\textcircled {1}$ to vacuum pump; $\textcircled {2}$ to gas cylinder; $\textcircled {3}$ pressure transducer output, to the data acquisition system; $\textcircled {4}$ Pt100 for measurement and control of temperature, to the data acquisition system and PID controller; $\textcircled {5}$ actuated ball valve; $\textcircled {6}$ pressure vessel; $\textcircled {7}$ electric heater input, PID controlled. (**c**) Mould with cylindrical cavities, 8.3 mm in height and 23.2 mm in diameter each; (**d**) Foam samples obtained from the process.

The foam production process is schematized in Fig. 2a.

The cylindrical samples were obtained from neat PP preforms (disks), punch cut from sheets. The sheets were produced from the granules using a compression molding machine (model P300P, Collin Gmbh, Ebersberg, Germany) as follows. The mould was preheated at 200 °C and the granules as received were placed on a flat press table for 2 min without pressure on a polytetrafluoroethylene sheet. The pressure was then increased to 5 bar in 1 min, then to 10 bar in 1 min and to 20 bar in 1 min. After cooling (to 25 °C), the pressure is brought to ambient condition and the produced PP sheet was extracted.

The graded-density sample was produced as detailed in Iaccarino et al.^6^. Briefly, preforms were produced by assembling 1 mm-thick slabs cut from PP sheet, compression molded as previously described. Polypropylene (PP) was kindly supplied by Sinopec Zhenhai Refning & Chemical Company (Zhejiang Province, China) as granules (commercial product identified as E02ES). CO_2_ was used as blowing agent and was provided by SOL S.p.A. (Monza, Italy).

#### Foaming procedure

PP foams were produced from the preforms using a custom-made thermoregulated pressure vessel (model BC-1, High Pressure Equipment Co., Erie, PA, USA), described in more detail in the work by Marrazzo et al.^31^ and shown in Fig. 2b. The pressure vessel, 0.3 L in volume, was equipped with a PID thermoregulator (mod. 1850, Gefran S.p.A., Provaglio d’Iseo, Brescia, Italy) for the temperature control and a pressure transducer (TK, Gefran S.p.A., Provaglio d’Iseo, Brescia, Italy) for the pressure measurement. The pressure discharge system consists of a discharge ball valve (model 15-71 NFB, High Pressure Equipment Co., Erie, PA, USA) and an electromechanical actuator (model 15-72 NFB TSR 8, High Pressure Equipment Co., Erie, PA,USA).

For the production of foamed cylinders, disks were placed in a three-piece mould with cylindrical cavities of 8.3 mm in height and 23.2 mm in diameter (Fig. 2c). The moulds were placed in the pressure vessel and a temperature of 160 °C was reached under vacuum to melt the polymer. The temperature was then increased to 146.5 °C, where saturation with CO_2_ was carried out at 120 bar in 10 min (sorption time). Finally, the ball valve was opened, leading to foaming of thobtainedks in the mould, where the foam could be shaped to the cylindrical cavity and set by cooling. Using preforms of various initial sizes and volumes, foamed samples of various densities were obtained for further testing (Fig. 2d).

For the production of graded-density beam, the optimized preform was placed in a box-shaped mould (9 × 140 × 15 mm^3^) and subjected to the same foaming procedure described above, but the sorption time was 30 s, instead of 10 min.

### Density measurements and volumetric fractions calculation

The average density of the produced foams ($\rho _f$) was obtained by using the following equation:

4 $$\begin{aligned} \rho _f=\frac{m_a}{m_a-m_w}(\rho _w-\rho _a)+\rho _a \end{aligned}$$

where $m_a$ is the weight of the foam in air, $m_w$ is the weight of the foam in water, $\rho _a$ is the density of the air and $\rho _w$ is the density of the water. At room temperature ($23\pm 2\,^{\circ }$C), $\rho _a=0.00120\pm 0.00002$ g/cm^3^ and $\rho _w=0.9975\pm 0.0005$ g/cm^3^^32^. $m_a$ ($\pm\, 0.0005$ g) and $m_w$ ($\pm\, 0.0005$ g) were measured using an analytical balance (MS Semi-Micro Model, Mettler Toledo, Milan, Italy). The same procedure was adopted to measure the density of neat PP (i.e., unfoamed PP, C6), denoted as $\rho _p$.

Then, the volumetric fraction of the polymer, $\chi _p$, can be computed as follows:

5 $$\begin{aligned} \chi _p=\frac{\rho _f-\rho _a}{\rho _p - \rho _a}, \end{aligned}$$

The absolute error on the measured foam densities, $\delta _{\rho _f}$, and the absolute error on the calculated volumetric fractions of the polymer, $\delta _{\chi _p}$, were obtained as described in the “Appendix”.

The parameters characterizing the cylindrical shaped samples, referred as C1–C7 and T1–T6 are reported in Table1.

**Table 1:** Foam samples parameters: sample name, density, density absolute error, volumetric fraction of the polymer.

Samples													
Name	C1	C2	C3	C4	C5	C6	C7 (neat)	T1	T2	T3	T4	T5	T6
$\rho _f$ [kg/m^3^]	71.6	95.5	112.5	118.0	149.7	181.2	902.5	80.9	112.7	177.4	300.7	383.6	532.7
$|\delta _{\rho _f}|$ [kg/m^3^]	0.2	0.2	0.2	0.2	0.3	0.3	3.1	0.4	0.3	0.3	0.5	0.5	0.6
$\chi _p$ [−]	0.0781	0.1046	0.1235	0.1295	0.1684	0.200	1	0.0885	0.1238	0.196	0.332	0.424	0.590
$|\delta _{\chi _p}|$ [−]	0.0004	0.0006	0.0007	0.0007	0.0008	0.001	–	0.0007	0.0007	0.001	0.002	0.002	0.003

### Measurement system

**Figure 3:**
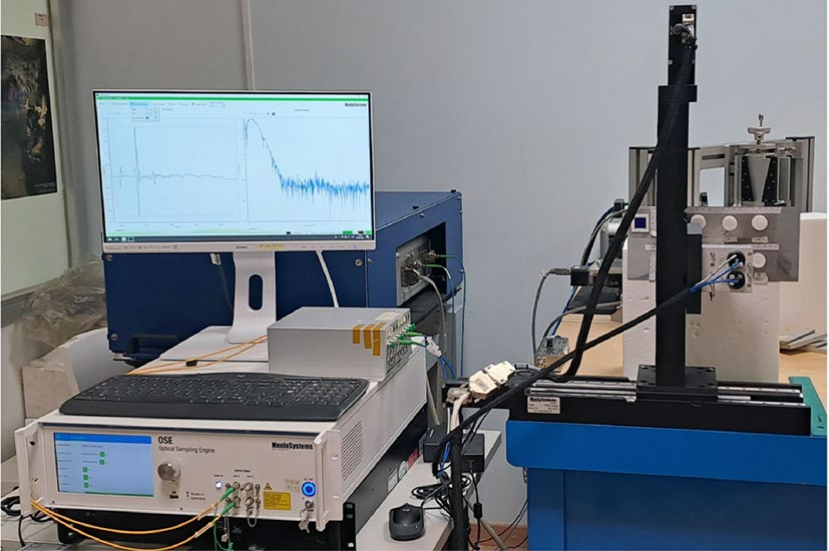
The adopted measurement set-up, with the optical sampling engine module (on the left side), whose output are connected to the THz reflection head positioned in front of the samples (right side). The sample are positioned on a flat metallic plane.

THz data have been gathered by means of a custom time-domain system developed by MenloSystems^33^. This device generates and gathers broadband THz pulses and enables measurement in both transmission and reflection modes. Figure 3 shows the system arranged for the reflection measurements mode exploited in this study.

The core of the system is the optical sampling engine (OSE) module, which exploits asynchronous optical sampling technique to perform high-speed scanning over some nanoseconds of time delay without using a mechanical delay line^34^, pushing the spectral resolution to the region of hundreds of MHz^35^. The OSE module includes two pulsed femtosecond lasers where one ultrafast laser delivers the pump pulse for the emitter antenna and the other laser delivers the probe pulse for the detector antenna. The lasers operate at a locked repetition rate with a tunable difference, providing the optical pulses for THz emission and measurement. The laser pulses are delivered via optical fiber to the THz photoconductive antennas (PCA), specifically the TERA15-TX-FC and TERA15-RX-FC fiber-coupled photoconductive antennas are used. The PCA are inserted in a compact THz reflection head with integrated optics for high-performance measurements, which is mounted on imaging stages equipped with stepper motors. These motors enable movement along the x-axis and the y-axis, resulting in a scan area of 300 mm $\times$ 300 mm and a minimum measurement spatial offset of 120 $\mu$m. The object under test has been positioned at the focal distance, i.e. at about 3 mm from the reflection head for the case at hand.

At each measurement point, the system collects signals within a 100 ps observation time window, which can be moved along a time scan range of 1 ns. The maximum depth that can be investigated for a 100 ps observation time window is $d_{max} < 100 \cdot 10^{-12} \cdot \ v/2$. This means that in the most favorable cases $( c \sim v)$ $d_{max}$ is less than 1.5 cm. Although the nominal bandwidth of the system is about 4 THz, in the case of normal reflection measurement and uncontrolled environmental conditions, the actual frequency range is $B = [0.2 - 2]$ THz. This means that, when the propagation occurs in air, the expected range resolution is $\Delta _r= \frac{c}{2B}=83.3 \upmu$m.

### Measurement procedure

The measurements procedure is summarized in the following steps:

1. The samples are placed on a metal plate at the focal distance from THz reflection head. The scanning area is 30 mm $\times 30$ mm for samples C1-C7 and T1–T6 and 100 mm $\times 30$ mm for the graded-density sample. The spatial offset along *x* and *y* axis is 0.5 mm for C1–C7 and T1–T6 and 0.25 mm for the graded-density sample. At each measurement point, the stored waveform is the average of those measured in a time window of 1 s.
2. The raw data are processed by means of an ad-hoc filtering strategy^36^. First, a frequency band-pass filter (BPF) procedure is applied to remove low- and high-frequency signal components, by picking signal components in the actual [0.2–2] THz range. Then, a noise filtering procedure, based on the singular value decomposition (SVD) of the band pass filtered data matrix, filters out further noise components, without affecting the spatial resolution.
3. The ToF $t_{M}$ is estimated by considering a generic processed waveform referred to a measurement point external to the sample and by looking for the time position of the maximum amplitude of the waveform, i.e. the peak due to the reflection by the metal (note that the metal is flat and parallel to the measurement aperture);
4. The ToFs $t_{S}$ and $t_{SM}$ are estimated for each measurement point $(x_p,y_p)$ belonging to the sample by searching the time positions at which the processed waveform exhibits its maximum amplitude values before and after $t_{M}$. The maximum amplitude occurring before $t_{M}$ represents the peak due to the air-sample interface and its time position is $t_{S}$, the other maximum amplitude is the peak due to the sample-metal interface and its time position is $t_{SM}$.
5. The ToFs $t_{S}$, $t_{SM}$, and $t_{M}$ are used to compute the thickness value $\Delta (x_p,y_p)$ by means of Eq. (2) and the effective refractive index $n_{eff}$ through Eq. (3).

### Calibration curve and THz density maps

To turn the effective refractive index into the density map the relationship between these quantities is experimentally established by analyzing the uniform density samples C1–C7, which show different and known density values, ranging from very low values up to that of neat PP.

To verify the measurement repeatability and the possible dependence of the results on the probed sample side, the samples have been analyzed by surveying both the largest sides and two data sets were collected for each side. Hence, for each sample, 4 effective refractive index and thickness maps were obtained, $\Delta _i(x_p,y_p)$ and $n_{eff,i}(x_p,y_p)$ with $i=1,..,4$.

The average effective refractive index $n_{avg}$ was estimated as follows. For the *i*-th dataset, $n_{eff,i}(x_p,y_p)$ was averaged among all measurement points to obtain the effective refractive index characterizing the dataset. Then, $n_{avg}$ was obtained as the average among the four values and associated to the density value $\rho _f$ corresponding to the sample at hand, as taken from Table 1. The average thickness $\Delta _{avg}$ was computed using the same procedure.

The calibration curve was then obtained performing a linear fitting of the average effective refractive index $n_{avg}$ estimated for all samples C1–C7, taking into account the standard deviation $\sigma _n$ arising from the computation of $n_{avg}$ for each sample.

The calibration curve is coherent with the relationship predicted by the medium theory of Scheller et al.^29^. Moreover, it has been validated against the T1–T6 samples, to check the matching between the density as estimated from their average effective refractive index and the one measured in “Density measurements and volumetric fractions calculation” section. Also for these samples, four data sets are collected (two for each side).

For the T1–T6 samples, the density maps obtained from the effective refractive index by using the calibration curve are inspected to show the capability of THz ToF imaging of characterizing the spatial variability of the foam density.

### Validation on the graded-foam sample

The graded foam sample has been analyzed with the proposed THz approach and the achieved density map has been compared with the result obtained with XRM.

XRM scans were obtained via X-ray microscope (SKYSCAN 1272, Bruker, Belgium). For the sample, 13600 scans (in the y direction, $1600 \times 3520$ pixels each) of its y-z cross-sections were collected, having a voxel size of $6.5 \times 6.5 \times 6.5$ μm^3^. For each scan, the voxel intensity scale (0–255 scale) was converted into the PP average density scale (0–902.5 kg/m^3^).

To compare with the THz result, an XRM density map in the x–y plane was built using MATLAB^®^ through the following steps. First, each XRM scan was averaged (simple mean) in the z direction, so that a vector $1\times 3520$ was obtained, corresponding to an x-coordinate line, for each y position. Then, all vectors were stacked into a $13600 \times 3520$ to obtain the density map in the x–y plane. Finally, the spatial resolution of this map was reduced to match the THz one, by averaging it with a square tessellation of $38 \times 38$ pixels, corresponding to 0.25 mm^2^ (i.e., the measurement offset adopted to perform the THz survey).

## Results

### Calibration curve

Table 2 reports the values of the average thickness $\Delta _{avg}$ and the average effective refractive index $n_{avg}$ for the calibration samples C1–C7. For each sample, the standard deviation expresses the variability among the four datasets.

**Figure 4:**
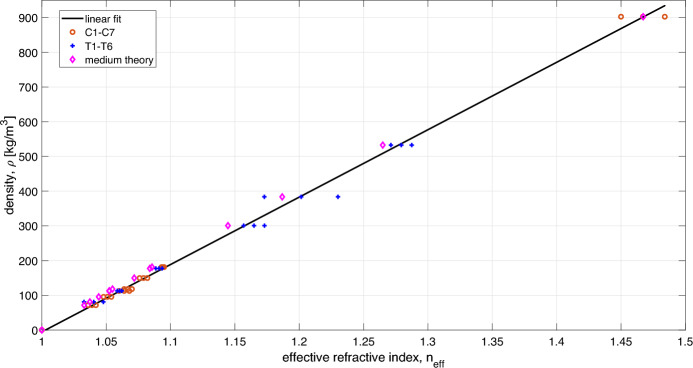
Calibration curve.

**Table 2:** Average thickness and effective refractive index estimated for the calibration samples C1–C7. The standard deviations are also reported.

Sample	C1	C2	C3	C4	C5	C6	C7
$\Delta _{avg}$ [mm]	9.0111	9.1945	9.3939	9.3186	9.5037	9.7544	1.9680
$|\sigma _\Delta |$ [mm]	0.0097	0.0573	0.0064	0.0079	0.0489	0.1032	0.0007
$n_{avg}$	1.039	1.051	1.064	1.067	1.079	1.094	1.467
$|\sigma _n|$	0.003	0.003	0.004	0.003	0.003	0.001	0.017

The average effective refractive index values were used to build the $n_{eff}-\rho$ calibration curve shown in Fig. 4, obtained via linear regression, including air in the data-fitting as the origin point for this curve ($\rho$ = 0, $n_{eff}$ = 1). In the plot, the data points with their standard deviations are reported as red circles, while the data points predicted by the medium theory^29^ are represented by the magenta diamonds. Note that the mixing formula given by Scheller et al.^29^ has been implemented considering PP as host medium and air spherical inclusions.

The relationship between $\rho$ and $n_{eff}$ resulting from the regression is given by:

6 $$\begin{aligned} \rho = 1941.9 n_{eff} - 1947.5 \end{aligned}$$

For data points, the absolute percentage error on the estimated density corresponding to the standard deviation is obtined as:

7 $$\begin{aligned} err = 100*1941.9 \sigma _n/\rho _f, \end{aligned}$$

with $\rho _f$ taken from Table 1. The values of *err* are reported in Table 3.

Table 4 reports the average refractive indices $(n_{avg})$ estimated for samples T1–T6 with the standard deviations $(|\sigma _n|)$, the corresponding estimated densities (\tilde{\rho }), the absolute error on the estimated density with respect to the nominal value $(|\rho _f-\tilde{\rho }|)$, the corresponding percentage error (*err*), the refractive indices corresponding to the nominal densities $(\hat{n}(\rho _f))$ according to Eq. (6) and the absolute discrepancy between the estimated $n_{avg}$ and $\hat{n}(\rho _f)$ $(|n_{avg}-\hat{n}(\rho _f)|)$. The average refractive indices estimated for samples T1–T6 are reported in Fig. 4 with their standard deviations as blue crosses.

**Table 3:** Absolute percentage error for the estimated density for samples C1–C7.

Sample	C1	C2	C3	C4	C5	C6	C7
*err*	$8.1 \%$	$6.1 \%$	$6.9 \%$	$4.9 \%$	$3.9 \%$	$1.1 \%$	$3.7 \%$

**Table 4:** Test samples T1–T6: average refractive index, standard deviation, average density resulting from the calibration curve, absolute error on average estimate density, absolute percentage error corresponding to the standard deviation, average refractive index resulting from the calibration curve using the nominal density in Table 1, absolute error on refractive index.

Sample	T1	T2	T3	T4	T5	T6
$n_{avg}$	1.0403	1.0604	1.0910	1.1649	1.2015	1.2793
$|\sigma _n|$	0.0075	0.0017	0.0024	0.0081	0.0286	0.0081
$\tilde{\rho }$ [kg/m^3^]	72.7	111.7	171.1	314.6	385.7	536.8
$|\rho _f-\tilde{\rho }|$ [kg/m^3^]	8.2	1.0	6.3	13.9	2.1	4.1
*err*	20%	3.0%	2.7%	5.0%	14.4%	2.9%
$\hat{n}(\rho _f)$	1.0445	1.0609	1.0942	1.1577	1.2004	1.2772
$|n_{avg}-\hat{n}(\rho _f)|$	0.0042	0.0005	0.0032	0.0072	0.0011	0.0021

**Figure 5:**
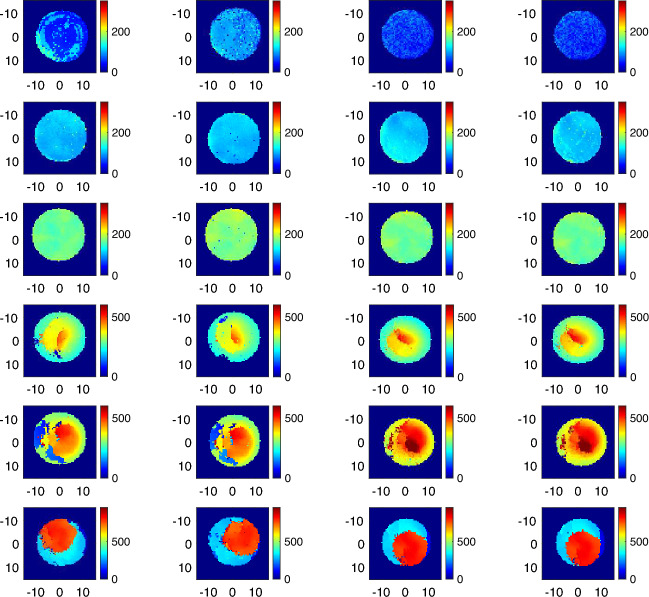
Density maps of T1–T6 samples. The samples with similar averaged density are shown in the same colorscale. The samples are arranged row-wise from top T1, T2, T3, T4, T5 and T6. Columns 1–2 correspond to Side 1, columns 3–4 correspond to Side 2. Axis dimensions are in mm.

Figure 5 shows the density maps for samples T1–T6 (two for each side of each sample) achieved turning the effective refractive index $n_{eff}$ in each measurement point into the corresponding density as given by the Eq. (6).

**Figure 6:**
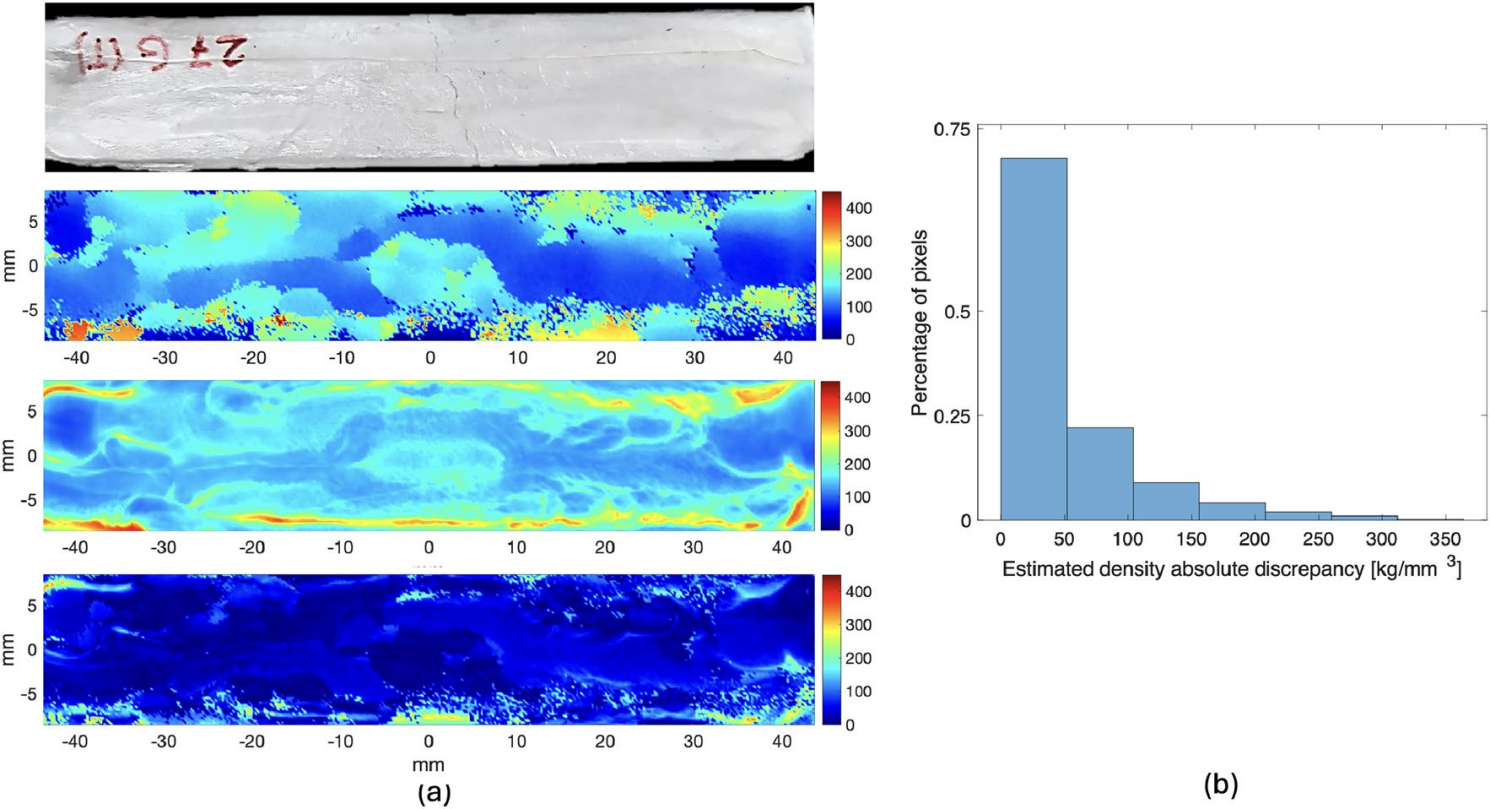
The graded-density foam sample. (**a**) from top to bottom: the sample; density map achieved via THz ToF imaging and using the calibration curve; density map achieved via X-ray microscopy; absolute discrepancy between the two maps. (**b**) Classification of the pixels of the absolute discrepancy map.

Figure 6 reports the results for the graded-foam sample. The figure shows the picture of the sample, the density map achieved via THz ToF imaging and using the calibration curve $\rho _{THz}$; the density map achieved via X-ray microscopy $\rho _{XRM}$ and the absolute discrepancy between the two maps, $\delta _\rho =|\rho _{THz}-\rho _{XRM}|$. The figure also shows the histogram of the classification of the pixels of $\delta _\rho$.

## Discussion

The relationship between the effective refractive index and the density has been built using samples C1–C7, which have been carefully manufactured in order to have a uniform density profile and morphology. The THz ToF analysis confirms that these samples are uniform, as their thickness and average effective refractive index exhibit a low standard deviation, with maximum standard deviations of 0.1 mm for the thickness and of 0.017 for the average effective refractive index, respectively.

The calibration curve is built using the estimated average effective refractive indices, revealing a linear relationship between the density and the effective refractive index in the range of interest, with a percentage error lower than $10 \%$. It is worth noting that the absolute error on the density has not been considered when fitting the calibration curve, as this is expected to be smaller than the accuracy which can be achieved using the THz ToF analysis.

The calibration curve has been validated with samples T1–T6. The estimated $n_{avg}$ are in very good agreement with the calibration curve, see Fig. 4. In particular, see Table 4, for samples T2, T3, T4 and T6, the estimated average densities agree with the nominal ones, with an absolute error lower than 10 kg/m^3^ and a percentage error on the estimated density of 5 %, which is consistent with the one observed for the calibration samples (C1–C7). To understand why samples T1 and T5 present larger errors, the THz retrieved density maps in Fig. 5, depicting the estimated density distribution resulting for each dataset measured for each sample, have to be observed. As expected, the density pattern of these samples is not uniform, but for T2 and T3. However, for all samples but T1 and T5, the images taken for the two sides are in very good agreement (but for a possible rotation of the sample). For T1 and T5 the two sides appear to be different, thus motivating the larger standard deviation in the estimated average refractive index and hence the larger error on the estimated density. It is worth noting that the agreement between the estimated average refractive index $n_{avg}$ and $\hat{n}(\rho _f)$, i.e., the refractive index resulting from the calibration curve using the nominal average densities in Table 1, is consistent with the standard deviation for all samples.

Besides corroborating the validity of the calibration curve, Fig. 5 allows to appreciate the capability of the proposed THz ToF imaging technique of providing a spatial map of the density within PP foams. Accordingly, the technique is able to retrieve manufacturing defects of samples, which seems uniform but are found to exhibit a non-uniform distribution of the density.

The analysis of the graded-foam sample confirms the capability of the proposed THz approach of mapping a very complex profile. As can be noticed from Fig. 6, the density maps obtained via XRM and THz, respectively, are in very good qualitative agreement. The quantitative agreement expressed in terms of $\delta _\rho$ also appears satisfactory, as more than 70% of the pixels have an error below 50 kg/m^3^. This is a very good agreement considering that the two images are not co-registered and the data have been acquired in different times and conditions. Moreover, it has to be taken into account: (1) the very complex distribution of the material in the direction of THz wave penetration; (2) the assumptions made for the volume averaging of both density-mapping procedures. Hence, we can conclude that the achieved result confirms that the proposed THz procedure is effective for a fast characterization of optimized foams, that is, foams with well defined 3D density maps.

## Conclusion

A simple THz ToF imaging approach for estimating the complex density profile of graded PP foams has been proposed. A relationship has been experimentally obtained to link the refractive index value to the density and it has been exploited to achieve a detailed map of the density pattern of uniform and not-uniform samples from THz measurements of the effective refractive index. The proposed approach provides a 2D map, which accounts for volumetric density changes and not only for the surface ones, without requiring knowledge of the point-by point thickness of the sample under test. The effectiveness has been proved by studying the complex density distribution of an optimized 3D sample and by comparing THz results with those obtained using X-ray microscopy. The results exhibit a good qualitative and quantitative agreement.

Notably, while the case of PP foams has been considered, the approach can be immediately extended to other types of plastic foams and, being simple and effective, it is easily exploitable in laboratories involved in the development of new foaming processes, as a non-invasive tool for manufacturing process control.

Future work regards the development of a data processing procedure based on an inverse scattering approach to retrieve the actual behavior of the refractive index along the propagation path.

## Data Availability

The datasets used and/or analyzed during the current study are available from the corresponding author on reasonable request.

## Appendix

Given Eq. (4), the absolute error on the measured average density of the foams was obtained as follows:

8 $$\begin{aligned} |\delta _{\rho _f}|=\rho _f\left( \frac{|\delta _{m_a}|}{|m_a|}+\frac{|\delta _{m_a}|+\vert \delta _{m_w}|}{|m_a-m_w|}+\frac{|\delta _{\rho _w}|+\vert \delta _{\rho _a}|}{|\rho _w-\rho _a|}\right) +|\delta _{\rho _a}|, \end{aligned}$$

where $\delta _{m_a}$, $\delta _{m_w}$, $\delta _{\rho _w}$ and $\delta _{\rho _a}$ are the absolute errors of $m_a$, $m_w$, $\rho _w$ and $\rho _a$, respectively.

Given Eq. (9), the absolute error of the volumetric fraction of the polymer was computed as follows:

9 $$\begin{aligned} |\delta _{\chi _p} |= \chi _p \left( \frac{|\delta _{\rho _f}|+\vert \delta _{\rho _a}|}{|\rho _f-\rho _a|}+\frac{|\delta _{\rho _p}|+\vert \delta _{\rho _a}|}{|\rho _p-\rho _a|}\right) , \end{aligned}$$

where $\delta _{\rho _p}$ is the absolute error of the measured density of the unfoamed PP.
